# Supplementary material for: Separable global and local beta burst dynamics in motor cortex of primates
Source: bioRxiv. 2025 May 10:2025.05.05.652217. Preprint. [Version 1] doi: 10.1101/2025.05.05.652217 (PMC12248081; doi:10.1101/2025.05.05.652217)
Supplement: Supplement 1 [file NIHPP2025.05.05.652217v1-supplement-1.pdf]

bioRxiv preprint doi: https://doi.org/10.1101/2025.05.05.652217; this version posted May 10, 2025. The copyright holder for this preprint (which was not certified by peer review) is the author/funder, who has granted bioRxiv a license to display the preprint in perpetuity. It is made available under aCC-BY 4.0 International license.

Supplemental Table 1: Days used for Behavioral, LFP, and spiking analysis:

Monkey H	Behavior (rewarded trials)	LFP	Spikes	Early /late	Monkey N	Behavior (rewarded trials)	LFP	Spikes	Early /late
Day 7	X (x)	X	X	Early	Day 7	X	X	X	Early
Day 8	X (x)	*	*	Early	Day 8	X (x)	X	X	Early
Day 10	X (x)	X	X	Early	Day 10	X (x)	X	X	Early
Day 11	X (x)	X	X	Early	Day 11	X (x)	**	**	Early
Day 13	X (x)	X	X	Late	Day 14	X (x)	X	X	Early
Day 14	X (x)	X	X	Late	Day 15	X (x)	X	X	Late
Day 16	X (x)	X	X	Late	Day 17	X (x)	X	X	Late
Day 20	X (x)	X	X	Late	Day 18	X (x)	X	X	Late
Day 21	X (x)	X	X	Late	Day 73	X (x)	X	****	Late
Day 23	X (x)	X	X	Late	Day 74	X (x)	X	****	Late
Day 24	X (x)	X	X	Late	Day 80	X (x)	***	****	Late
					Day 81	X (x)	***	****	Late
					Day 85	X (x)	***	****	Late
**Total sessions:**	**11**	**10**	**10**			**13**	**9**	**7**	

* Significant head movements that eventually broke the subcortical array connector and impaired spiking detection. Connector was repaired on Day 9.

** No neural data recorded during this day due to animal movements preventing headstage clipping

*** Subcortical array connector broken

**** No spiking activity detectable on cortical array during online behavior sessions

bioRxiv preprint doi: https://doi.org/10.1101/2025.05.05.652217; this version posted May 10, 2025. The copyright holder for this preprint (which was not certified by peer review) is the author/funder, who has granted bioRxiv a license to display the preprint in perpetuity. It is made available under aCC-BY 4.0 International license.

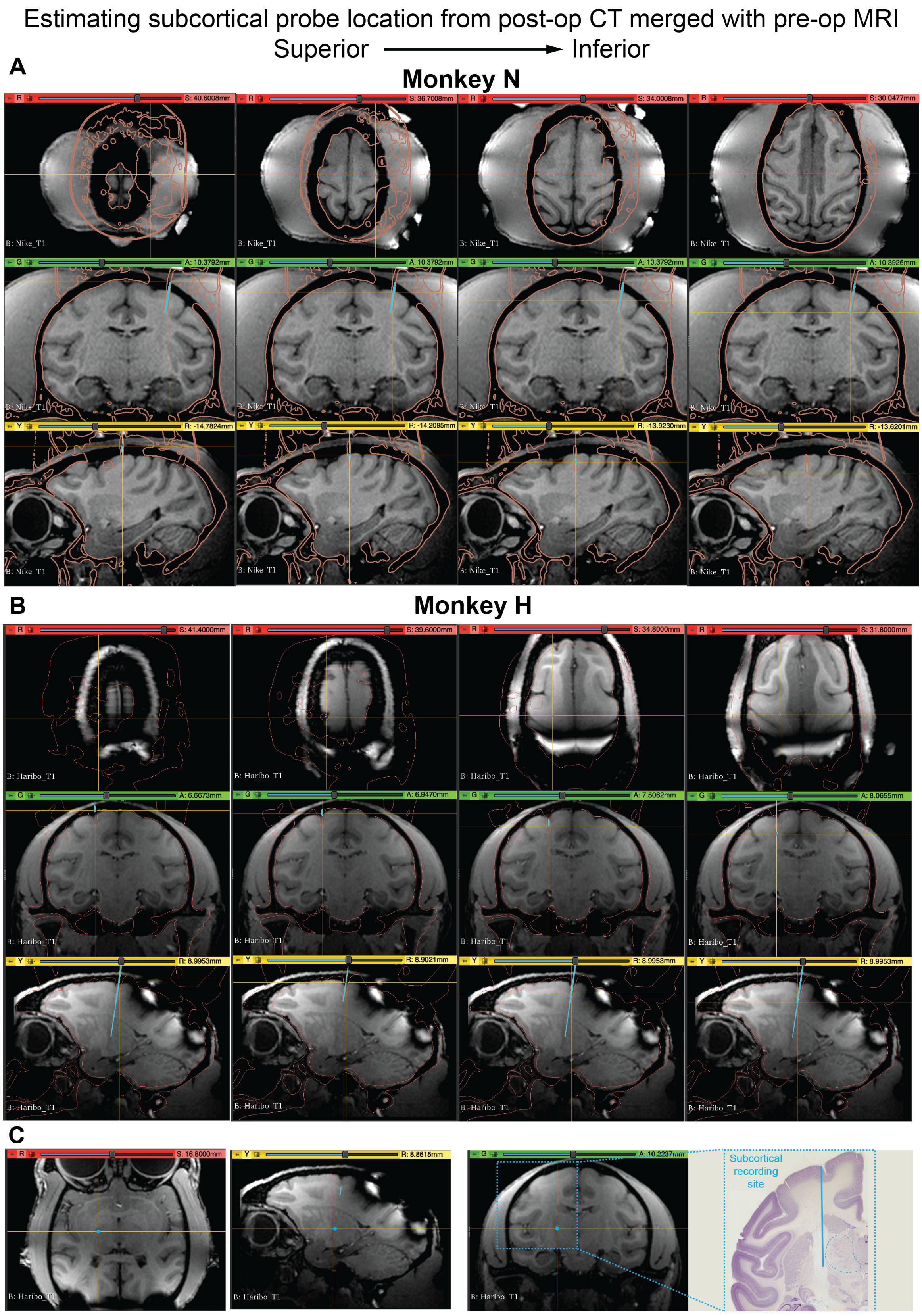

bioRxiv preprint doi: https://doi.org/10.1101/2025.05.05.652217; this version posted May 10, 2025. The copyright holder for this preprint (which was not certified by peer review) is the author/funder, who has granted bioRxiv a license to display the preprint in perpetuity. It is made available under aCC-BY 4.0 International license.

Supplemental Figure 1: Estimating the subcortical probe location

Prior to surgery, cranial 3T MRI images were acquired for both animals using a T1-weighted sequence. After surgery, cranial computed tomography (CT) images were acquired for both animals. The MRI images were aligned and resliced in ACPC coordinates using FieldTrip . Then, both the CT and re-sliced MRI images were loaded into 3D Slicer. The CT images were windowed using the default CT-bone window and were thresholded to extract a volume containing the skull. This volume also contained the implanted microelectrode array in perilesional cortex and support body of the subcortical electrode array.

A, B: The extracted skull and implanted electrode volume (CT, orange) was then manually aligned with the MRI such that the CT skull aligned maximally with the skull in the MRI image. When traversing the axial axis of the scan, the CT skull volume shows excellent alignment to the MRI in the axial, coronal, and sagittal axes in places where the skull was not disrupted due to the surgical intervention.

After alignment, a 3D model of the subcortical electrode array was imported into the 3D slicer image (cyan). The array was manually aligned such that the support body of the array maximally overlapped with the part of the CT volume corresponding to the support body. The silicon shank part of the subcortical electrode array was not visible on the CT. The alignment between the support body and the CT volume was verified by scanning through the axial, sagittal, and coronal axes and can be seen in **A, B**. The final location of the recording electrodes was then visualized in **C** (Monkey H), and **Fig 1D** (Monkey N).

T1-weighted sequences do not visualize subcortical nuclei particularly well. To estimate the structures where the subcortical electrodes were positioned, brain slices from the Brainmaps.org atlas were visualized[69]. Slices that best matched the cortical anatomy of the coronal MRI slice where the electrodes were placed (shown in C and Fig. 1D) were selected (Monkey N: Atlas slice 8.2mm AP, Monkey H: Atlas slice 10.0mm AP). These slices were within 1-2mm of the AP axis of the animals' MRI (Monkey N: 9.9mm AP, Monkey H: 10.2mm AP). The electrode trajectories were estimated on the atlas slices by computing the ML and DV coordinates of the final electrode position and the angle of the electrode tract in the MRI. The angle and the final electrode position dictated the electrode trajectory shown in Fig. 1D and **C.**

These slices reveal that the final electrode positions were either in (Monkey N) or just outside in the white matter (Monkey H), the VL nucleus of the motor thalamus. For the purposes of this study, we simply refer to these electrode recording locations as “subcortical”.

bioRxiv preprint doi: https://doi.org/10.1101/2025.05.05.652217; this version posted May 10, 2025. The copyright holder for this preprint (which was not certified by peer review) is the author/funder, who has granted bioRxiv a license to display the preprint in perpetuity. It is made available under aCC-BY 4.0 International license.

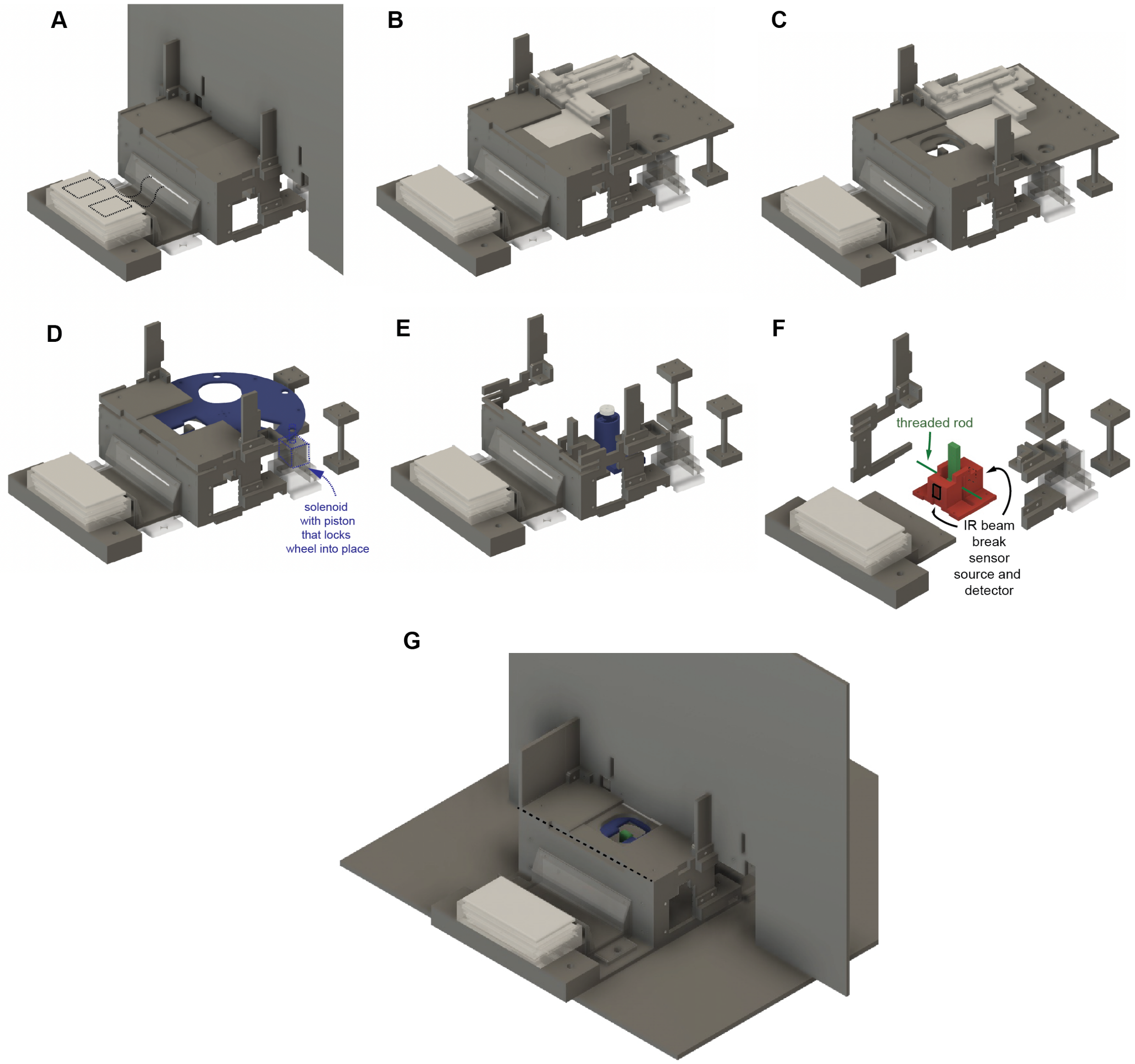

Supplemental Figure 2: Reach-to-grasp task

The CAD model of the reach-to-grasp task.

A) Appearance of the fully assembled reach-to-grasp task from the perspective of the side camera at the start of a trial. The button is highlighted in white. The button houses two square force sensitive resistors (Sparkfun.com, SEN-09376) with wires that are routed to the back of the task (indicated by dashed black lines). The wires are covered by a "wire protector" piece. Animals must first depress the button for a designated hold period in order to open the door to the slot and object to start the trial.

B) Removal of "backsplash" for better visualization of the slot door apparatus (shown in white). A linear slide potentiometer (Bourns PSM60-081A-103B2) is seated behind the backsplash and has a lever that is fixed to a 3D printed door.

C) After the button has been depressed for the designated hold period, the door slides open, revealing the object and the slot that must be reached through in order to grasp the object.

D) Removal of the door and the structural pieces of the task apparatus for better visualization of the "wheel" that has the slot cutouts. The wheel is fixed to a DC motor through a coupler (shown in E). Prior to each trial, the motor spins the wheel such that the correct slot is aligned with the object. A

bioRxiv preprint doi: https://doi.org/10.1101/2025.05.05.652217; this version posted May 10, 2025. The copyright holder for this preprint (which was not certified by peer review) is the author/funder, who has granted bioRxiv a license to display the preprint in perpetuity. It is made available under aCC-BY 4.0 International license.

magnetic solenoid's (Adafuit.com, Product ID = 3992) piston is released through holes on the outside of the wheel to lock the wheel in place and prevent the animal from moving the wheel during a trial. The tripod slot is shown in the current trial.

E) The wheel and other structural pieces of the task have been removed to visualize the DC motor (Adafuit.com, Product ID 4416), the DC motor coupler (Pololu Robotics and Electronics, Pololu item # 1079, Pololu Universal Aluminum Mounting Hub for 3mm Shaft, #2-56 Holes (2-Pack), and the housing that was 3D printed to attach the motor to the task apparatus.

F) Removal of the wheel motor and other structural parts to reveal the "object" (green), and the custom 3D printed apparatus that houses the object (red) and the IR beam break sensors that detect the object height. The object has a threaded rod that twists through its body, and through vertical slots on the housing. This rod and the track for the rod in the housing constrains the motion of the object to be up and down. The housing also contains cutouts for the IR beam break sensors (Adafruit.com, Product ID 2167) that detect if the object has been lifted above the 15mm height (shown in black).

G) View of the re-assembled task with the door open revealing the slot and the object to be lifted. The black horizontal line indicates a line used in behavioral analysis (Methods, Reach-to-grasp behavior, automatic extraction of behavioral timepoints, relevant to identification of the "crossing frame")

bioRxiv preprint doi: https://doi.org/10.1101/2025.05.05.652217; this version posted May 10, 2025. The copyright holder for this preprint (which was not certified by peer review) is the author/funder, who has granted bioRxiv a license to display the preprint in perpetuity. It is made available under aCC-BY 4.0 International license.

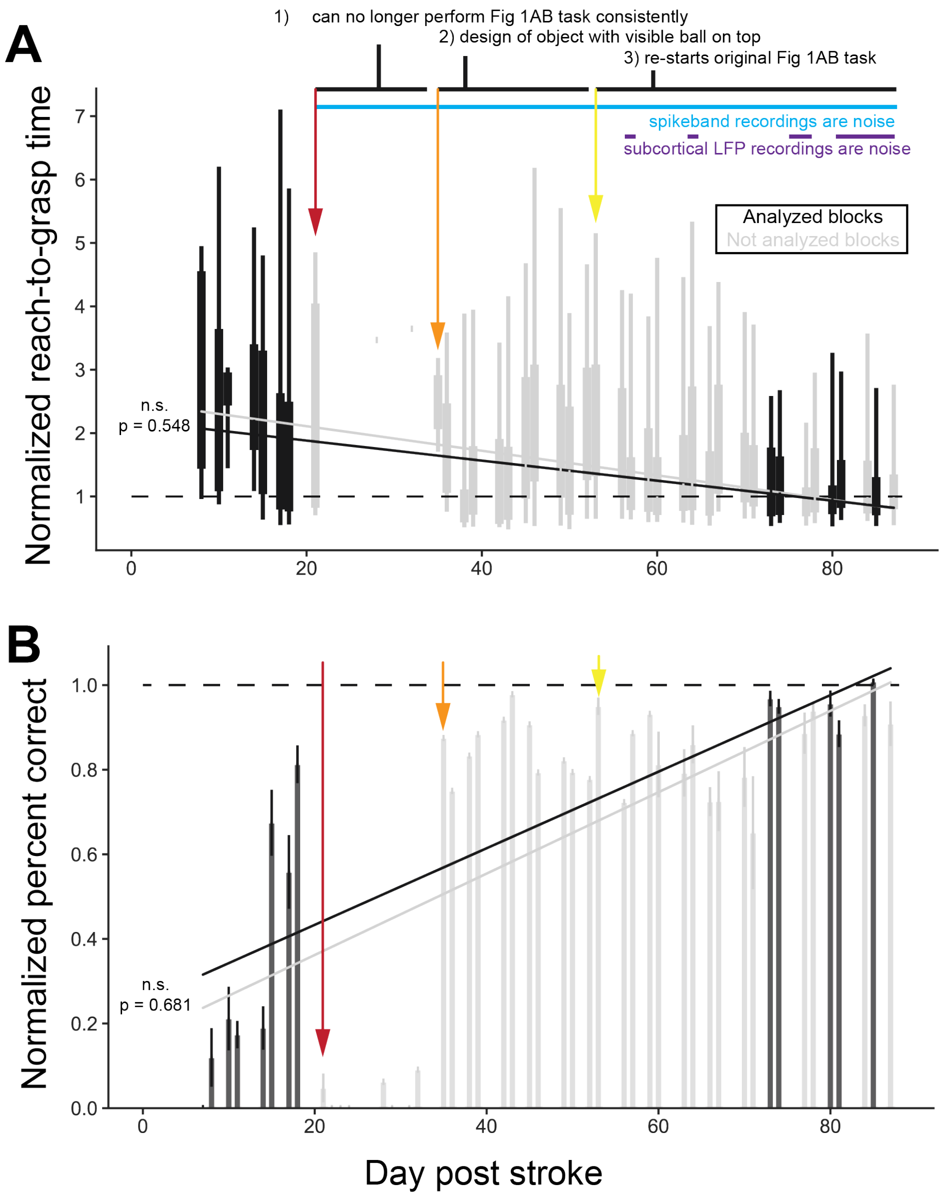

Figure S3: Monkey N full recovery curve

Monkey N underwent an unusual recovery curve. Initially, from days 7 to 18 after stroke, Monkey N exhibited a typical recovery with a reduction in reach-to-grasp time and an increase in percent correct. At Day 21 (red arrow), we noticed a return of his arm and hand impairment concomitantly with a significant reduction in cortical array spiking quality. We performed neuroimaging (CT) but were unable to see evidence of an obvious secondary brain injury or of an infection. We continued attempting to test Monkey N using the reach-to-grasp task outlined in Fig 1AB, but he continued to exhibit an inability to perform the task and poor cortical array spiking signals. At Day 35 (orange arrow) we introduced a new “object” within the same reach-to-grasp task format. The new object had a large ball on top of the existing rectangular object, which made finding the object and grasping it much easier. We continued testing Monkey N on this task until Day 53 at which point he was able to begin performing the original reach-to-grasp task again. We are still investigating the possible causes of his secondary decline.

Because of this unusual recovery curve and because we have been unable to pinpoint the cause of the secondary decline, we have chosen to focus on the initial albeit incomplete recovery curve (days 7-20) augmented by a few days at the end of his recovery once his performance was back at pre-stroke baseline. Since the purpose of our study is to examine how global and local beta dynamics change with

bioRxiv preprint doi: https://doi.org/10.1101/2025.05.05.652217; this version posted May 10, 2025. The copyright holder for this preprint (which was not certified by peer review) is the author/funder, who has granted bioRxiv a license to display the preprint in perpetuity. It is made available under aCC-BY 4.0 International license.

recovery from stroke, it was important to us that our choice of sessions did not modify the overall dynamics of Monkey N's recovery curve. To test this, we fit a linear regression to behavioral data from all days (gray lines in A, B) and fit a regression to only the sessions we analyzed in Fig 1EFG (black lines in A, B). We tested whether the slopes of these regressions differed significantly (F-test). For both normalized reach-to-grasp time and normalized percent correct there was no significant difference between the slopes of the gray and black lines (A: normalized reach-to-grasp time, $p = 0.548$, B: normalized percent correct, $p = 0.681$). See Table S1 for more details about exactly which days of data were used in each analysis.

bioRxiv preprint doi: https://doi.org/10.1101/2025.05.05.652217; this version posted May 10, 2025. The copyright holder for this preprint (which was not certified by peer review) is the author/funder, who has granted bioRxiv a license to display the preprint in perpetuity. It is made available under aCC-BY 4.0 International license.

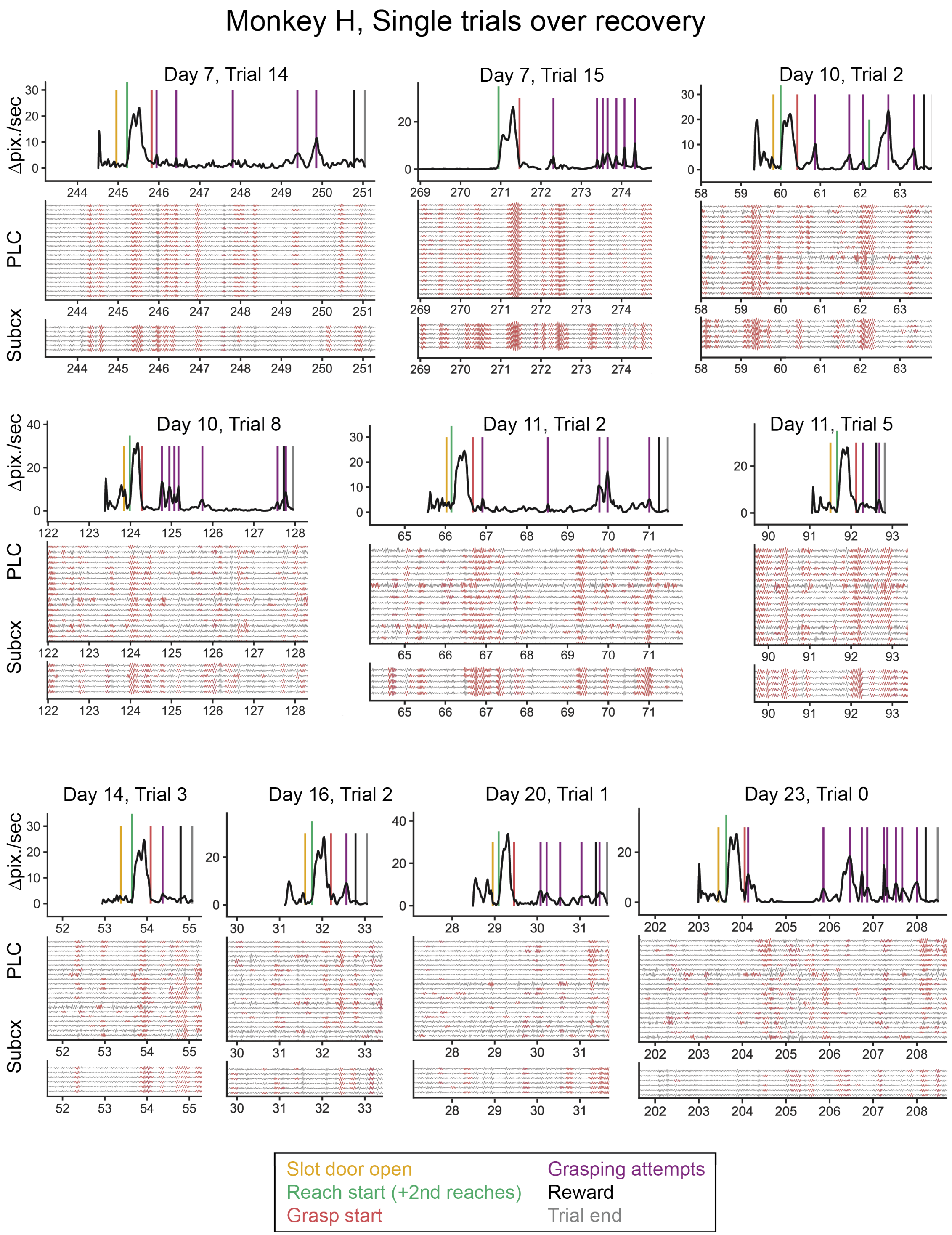

Figure S4: Monkey H single trials over the course of recovery. In the same style as Fig. 1JK.

bioRxiv preprint doi: https://doi.org/10.1101/2025.05.05.652217; this version posted May 10, 2025. The copyright holder for this preprint (which was not certified by peer review) is the author/funder, who has granted bioRxiv a license to display the preprint in perpetuity. It is made available under aCC-BY 4.0 International license.

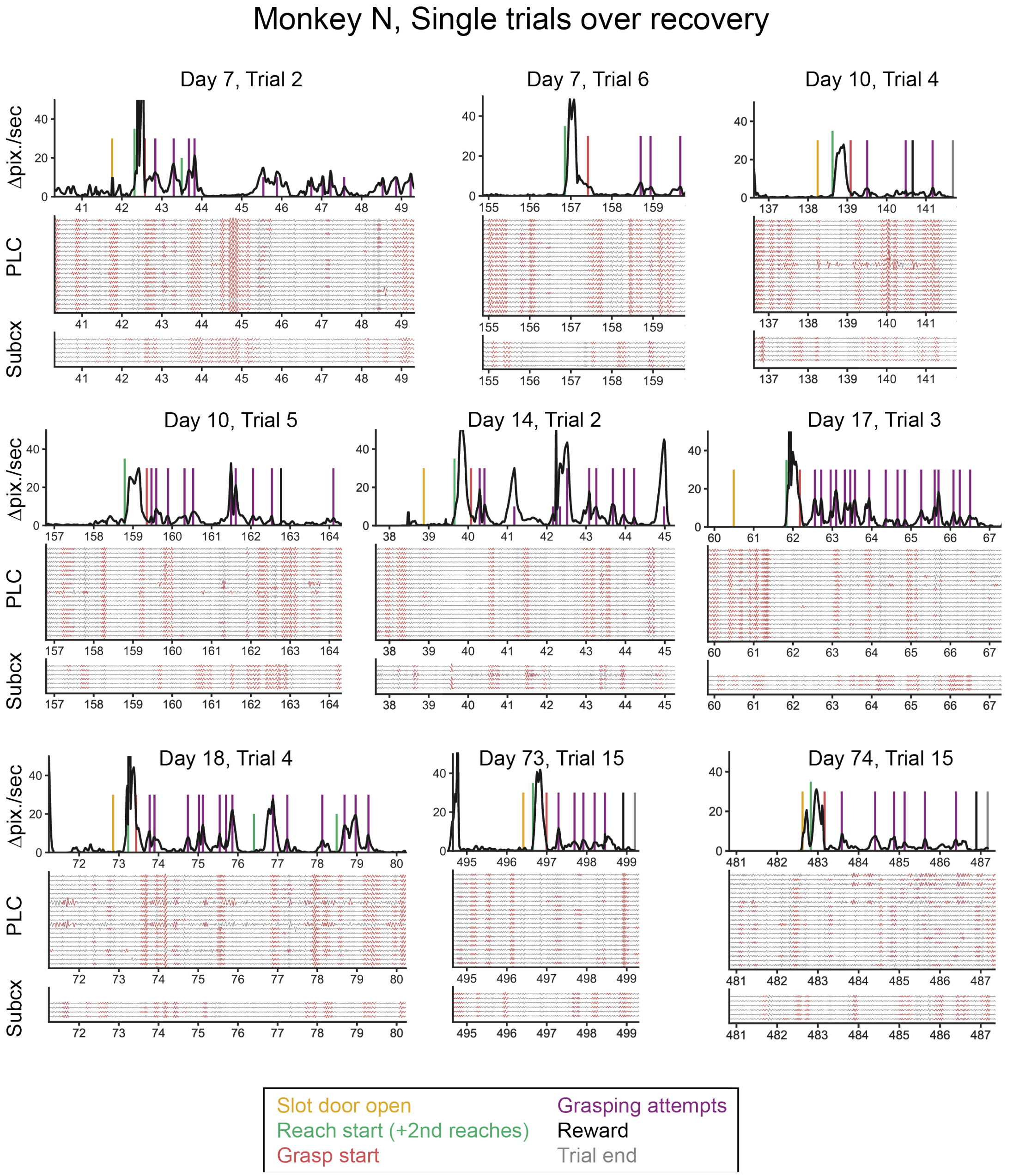

Figure S5: Monkey N single trials over the course of recovery. In the same style as Fig. 1JK.

bioRxiv preprint doi: https://doi.org/10.1101/2025.05.05.652217; this version posted May 10, 2025. The copyright holder for this preprint (which was not certified by peer review) is the author/funder, who has granted bioRxiv a license to display the preprint in perpetuity. It is made available under aCC-BY 4.0 International license.

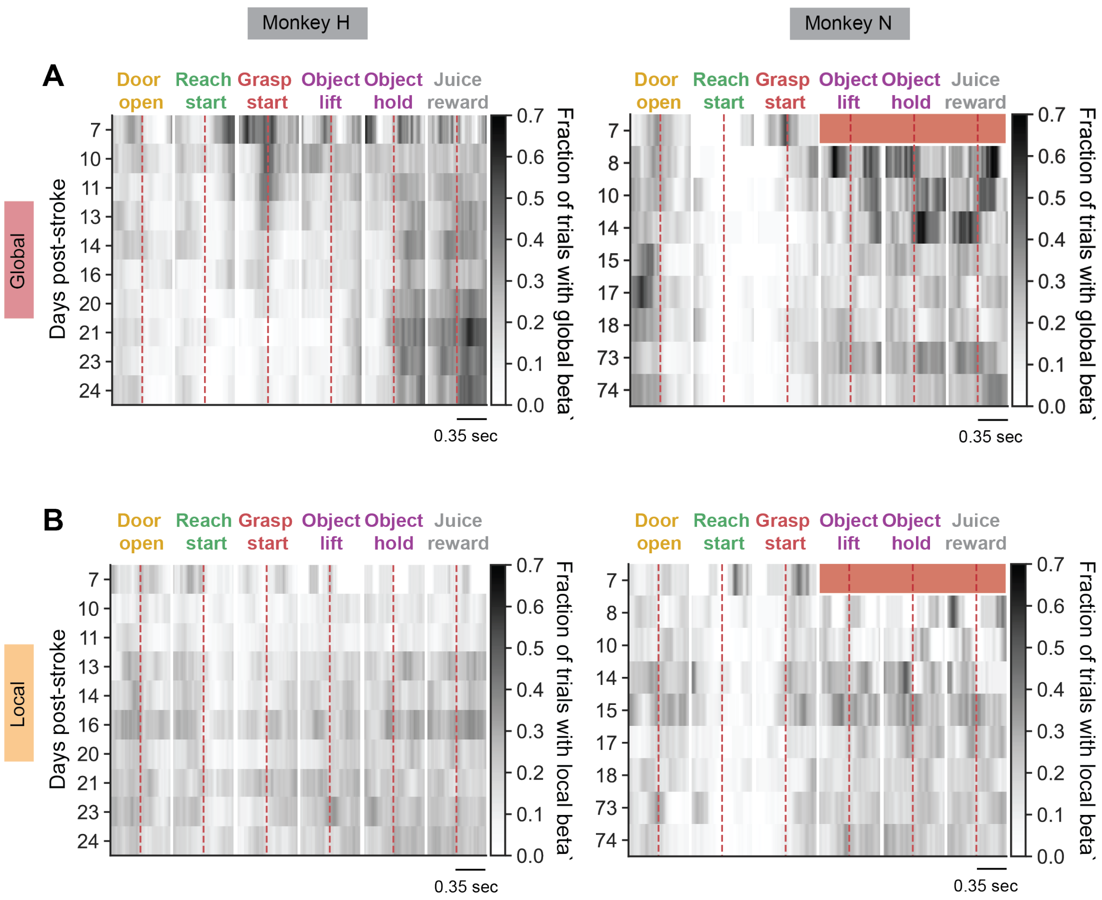

Figure S6. Alternative visualization of data in Fig 4AB. Individual post-stroke days are shown as rows, and darkness of the row indicates higher fraction of trials with global (A) or local (B). Red shading indicates no data present (no trials with successful lifts in Monkey N on day 7).

bioRxiv preprint doi: https://doi.org/10.1101/2025.05.05.652217; this version posted May 10, 2025. The copyright holder for this preprint (which was not certified by peer review) is the author/funder, who has granted bioRxiv a license to display the preprint in perpetuity. It is made available under aCC-BY 4.0 International license.

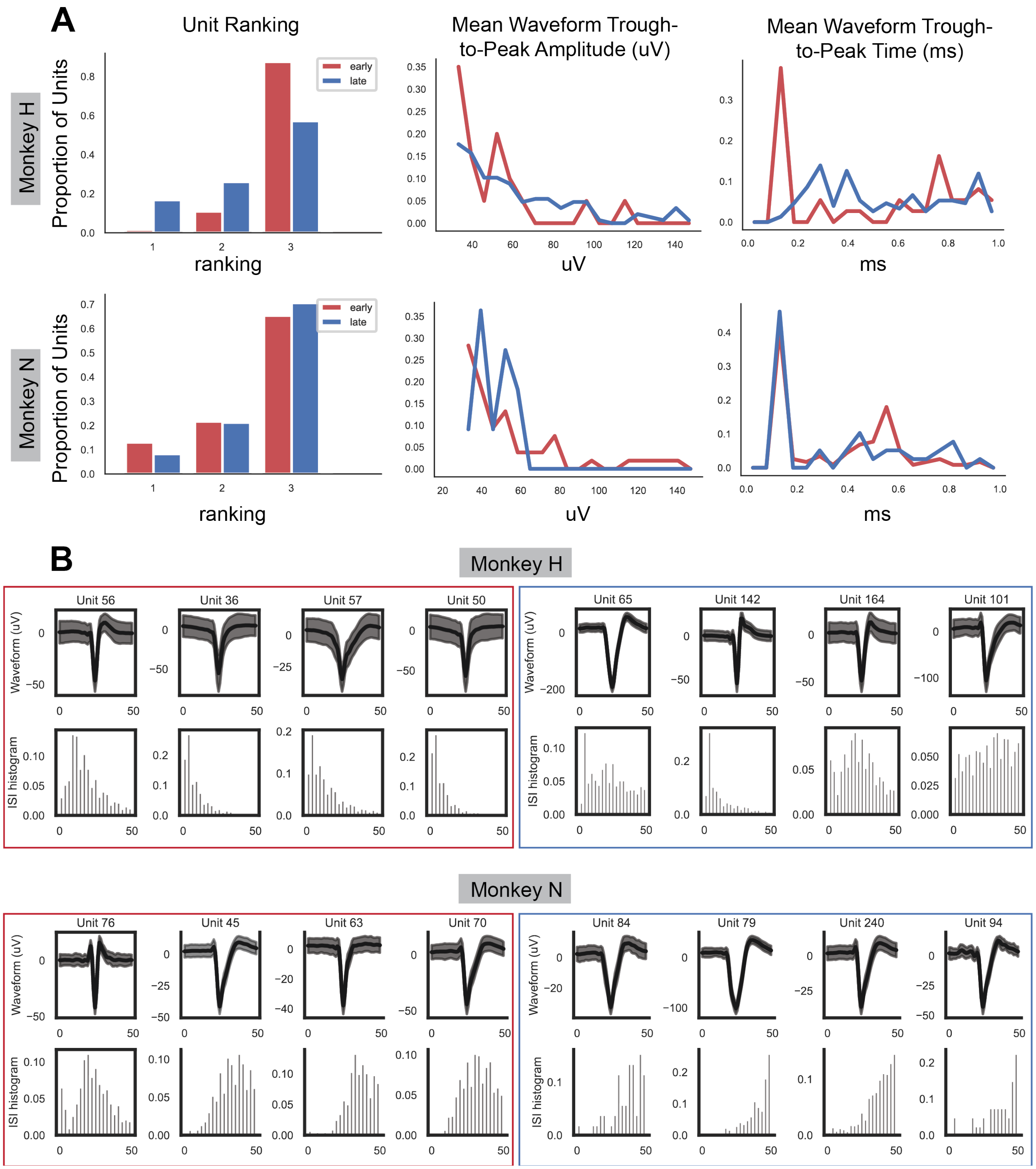

Figure S7. Properties of single units recorded during early recovery and late recovery. (A, *left*) Unit ratings during early vs. late recovery. Units were rated according to SNR: Rating 3: SNR > 3.0, Rating 2: SNR > 4.0, Rating 1: SNR > 8.0. Monkey N had very similarly ranked units early vs. late in recovery whereas Monkey H had a higher proportion of higher ranked units late in recovery. (A, *center*) Distribution of trough-to-peak amplitude (uV) for early vs. late units. Animals show trends in different directions: Monkey H has higher amplitude units later in recovery whereas Monkey N has higher amplitude units early in recovery. (A, *right*) Distribution of waveform widths (measured via trough-to-peak time) for animals. Monkey N has a very consistent distribution of waveform widths in early vs. late whereas Monkey H has a higher proportion of very short waveform widths early in recovery compared to late. (B). Examples of waveforms and inter-spike

bioRxiv preprint doi: https://doi.org/10.1101/2025.05.05.652217; this version posted May 10, 2025. The copyright holder for this preprint (which was not certified by peer review) is the author/funder, who has granted bioRxiv a license to display the preprint in perpetuity. It is made available under aCC-BY 4.0 International license.

interval histograms from randomly selected units from Monkey H and Monkey N during early (red box) and late (blue box) recovery.
